# Na,K-ATPase Expression Can Be Limited Post-Transcriptionally: A Test of the Role of the Beta Subunit, and a Review of Evidence

**DOI:** 10.3390/ijms25137414

**Published:** 2024-07-06

**Authors:** Elena Arystarkhova, Kathleen J. Sweadner

**Affiliations:** Department of Neurosurgery, Massachusetts General Hospital and Harvard Medical School, Charlestown, MA 02129, USA

**Keywords:** Na,K-ATPase, sodium pump, genetic disease, endoplasmic reticulum, biosynthesis, protein misfolding

## Abstract

The Na,K-ATPase is an α–β heterodimer. It is well known that the Na,K-ATPase β subunit is required for the biosynthesis and trafficking of the α subunit to the plasma membrane. During investigation of properties of human ATP1A3 mutations in 293 cells, we observed a reciprocal loss of endogenous ATP1A1 when expressing ATP1A3. Scattered reports going back as far as 1991 have shown that experimental expression of one subunit can result in reduction in another, suggesting that the total amount is strictly limited. It seems logical that either α or β subunit should be rate-limiting for assembly and functional expression. Here, we present evidence that neither α nor β may be limiting and that there is another level of control that limits the amount of Na,K-ATPase to physiological levels. We propose that α subunits compete for something specific, like a private chaperone, required to finalize their biosynthesis or to prevent their degradation in the endoplasmic reticulum.

## 1. Introduction

Na,K-ATPase is a membrane protein that transports three sodium ions out of the cell and two potassium ions into the cell and maintains the ion gradients that underlie many cellular processes. Its expression is controlled at multiple levels so that the capacity for ion transport matches the physiological needs of different tissues and cells [1,2]. The highest expression is in conducting tissues such as brain and heart and in tissues that secrete fluids. Up to three isoforms of both α and β, the major subunits, are expressed in different tissues, fine-tuned for different physiological roles, and there are seven small regulatory subunits from the FXYD gene family. The normal stoichiometry is 1α:1β:1FXYD. For a recent review, see [3,4].

There are human mutations of Na,K-ATPase ATP1A3 that produce neurological disease, and the mutations usually are dominant. Some amino acid substitutions, by virtue of destabilizing the protein, interfere with its biosynthesis even when it still has activity. We have been investigating the underlying causes of dominance in mutations of *ATP1A3*, the gene that encodes the α subunit that has properties adapted for excitable tissue such as neurons [5,6]. The experimental system employed here is a human cell line that expresses endogenous ATP1A1 (α1), endogenous β subunit (β1 and β3), and FXYD5 but negligible endogenous ATP1A3 (α3). Stable cell lines were made where WT α3 or α3 with individual mutations can be expressed under a tetracycline-inducible promoter. This sets the stage for assessment of the cell biological responses to mutated α3 in isogenic cells.

In brief, the dominance of mutations that cause the neurological condition known as rapid-onset dystonia-parkinsonism (RDP) was found not to be correlated with the amount of residual Na,K-ATPase activity [7], implying a gain-of-function effect. The dominance of two ATP1A3 mutations that cause very severe infantile disease was shown to be due to protein misfolding during biosynthesis, with concomitant proapoptotic signaling [7,8]. The dominance of mutations in Arg756, where ataxia and other symptoms manifest in childhood fevers associated with infections, was shown to be due to its temperature sensitivity in the febrile range, leading to ATP1A3 degradation [9]. The contrasting cell biological features of RDP and alternating hemiplegia of childhood (AHC) mutations are currently under investigation.

A key observation in these stable cell lines is that, when α3 is induced, the level of α1 is reciprocally reduced. Conversely, when an α3 mutation causes a reduction in its own biosynthesis, the level of α1 is less impacted than when expressing WT α3 [7,8,9]. It appears that, during biosynthesis, α1 and α3 may be in competition for a limiting factor. In the discussion, we review 12 prior studies that can be interpreted in this light.

It is well established that Na,K-ATPase α and β subunits associate tightly in the endoplasmic reticulum during biosynthesis in the ER [10,11,12]. Furthermore, it has not been possible to separate α from β and reassemble them again after biosynthesis or to demonstrate exchange of β subunits among α–β dimers in the membrane. However, α and β do not associate with each other in the ER until the last third of the α subunit has been translated [13,14]. This was explained by crystal structures of purified Na,K-ATPase that showed that the major α–β contact surface of α is the extracellular loop between the seventh and eighth transmembrane spans. The major contact surface of the β subunit is composed of four sections that are widely separated in its primary sequence, and so β must be folded before binding to α [15,16]. Within the lipid membrane, the single transmembrane span of β lies almost parallel to α’s seventh transmembrane span (M7). These α–β interactions are thought to complete the folding of α subunit by stabilizing the membrane association of M7–M10, which is otherwise unstable [17]. Both subunits were shown to first interact with BiP (GRP78), a lumenal analog of the cytoplasmic chaperone HSP70, which protected them from degradation until assembly occurred [18]. It has also been shown that nascent α without β interacts with β-COP, part of the COP-I complex, which retains it in the ER [19]. β-COP was proposed to interact with a prominently displayed dibasic motif on the surface of the Na,K-ATPase A domain (the domain closest to the N-terminus), which is the first domain to fold during biosynthesis.

The β subunit is essential for the maturation and trafficking of the α subunit because only β has N-linked glycosylation sites, which are used by a complex system for determining whether a protein in the ER is folded and can proceed to the Golgi apparatus [20]. The scheme that emerges is that α and β are made separately, interacting with chaperones and retention mediators, until the appropriate contact surfaces on α and β are available for interaction to occur, accompanied by the release of BiP. Calnexin, a critical ER chaperone that tracks the progress of folding of glycoproteins, can then assume its role to determine whether the αβ complex should go on to the Golgi apparatus or be degraded [19].

The hypothesis tested in the present work was whether α1 and α3 compete for a limiting amount of β subunit during biosynthesis. This was conducted by providing the cell with an excess of β and examining the consequences. Because we are using 293 cells to model the effect of α3 mutations in neurons, we manipulated the neuronal β1 subunit to test the hypothesis.

## 2. Results

### 2.1. Characteristics of the Expression System

According to a transcriptomics study [21], HEK293 cells express Na,K-ATPase α1 subunit mRNA most abundantly, and endogenous α3 mRNA is present at only 2% of the level of α1. The level of α1 mRNA was 50–60% higher than β1 and β3 mRNAs combined. There were negligible levels of α2, β2, α4 (only in sperm), or closely related H,K-ATPase mRNAs. β3 mRNA was ~5 times the level of β1 mRNA but, in the present experiments, we followed only β1 because, first, it is the most abundant naturally occurring partner of α3 and, second, β3 is not expressed in neurons, where human mutations of α3 have their functional impact. The relative amounts of β1 and β3 protein in 293 cells are not known, and it is normal for there to be disparities between mRNA and protein levels in Na,K-ATPase [1].

The aim was to determine if Na,K-ATPase β subunit is rate-limiting for the expression of either endogenous α1 or induced α3 in the expression system we have used to compare the properties of disease-causing ATP1A3 mutations. The salient properties of the system are that, when expression of α3 is turned on, its mRNA levels reach maximum levels within 48 h. α3 protein appears within 24 h and doubles between 24 h and 48 h. The cells double every 30 h and, so, the relative levels of α1 and α3 are a combination of new protein synthesis and turnover of the α1 present in the cells when tetracycline is first added [7].

A feature of this cell line is that the level of total α subunit appears to be regulated such that α1 levels go down when α3 levels go up [7,8,9]. A pan-specific anti-α antibody shows a nearly constant level of expression. Any elevation of protein reacting with the pan-specific antibody represents an increase from the normal physiological state.

### 2.2. Transient Transfection of β1

**Figure 1:**
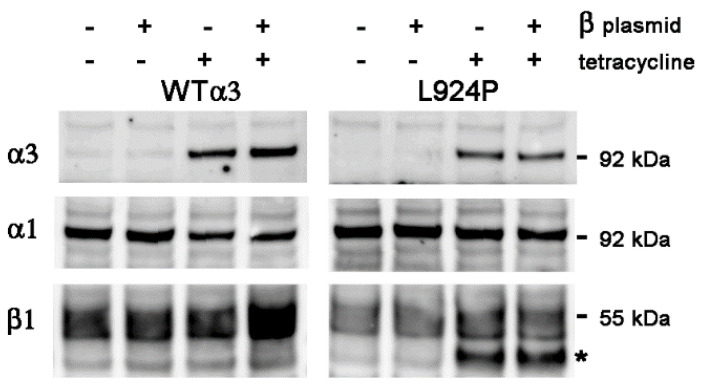
Transient overexpression of β1 for 24 h. In the absence of both β1 plasmid and tetracycline, the small amount of stain for α3 is presumably the low level of endogenous expression. The presence of β1 plasmid without tetracycline induction did not alter expression, as expected. The host cell line expressed α3 when tetracycline was added, also as expected, and the level of α1 was correspondingly reduced, as reported before. This was true for both WT α3 and the L924P α3. As previously reported, L924P misfolds during biosynthesis and retains some of the β1 subunit in the ER. With both β1 plasmid and tetracycline, there was a modest increase in β1 subunit at 24 h. The asterisk marks the faster-migrating β1 with high mannose oligosaccharides. Our hypothesis was that α1 levels should rise to at least the level seen in the absence of tetracycline if endogenous β1 is limiting. This was not seen in either the WT or the mutant.

The vector for expression of excess β1 was pcDNA4/TO/human β1, which has zeocin resistance for selection and a tetracycline sensitive promoter for β1. Pilot experiments were conducted with transient co-transfection of the β1 vector with pcDNA6/TR plasmid into two stable α3 recombinant cell lines, one with WT α3 and the other with L924P. The cells were allowed to recover for 24 h before the addition of tetracycline to simultaneously turn on expression of both β1 and α3. Figure 1 (left) shows results 24 h after addition of tetracycline, where the α3WT cells were either controls with no β1 plasmid or sister cultures after transient transfection. In the top panel, expression of α3 was strongly dependent on the presence of tetracycline and modestly increased with the β1 plasmid (60% increase at this timepoint). In the bottom panel, as expected, there was an increase in β1 only when both plasmid and tetracycline were present (76% increase). In contrast to our expectations, the expression of α1 was not increased with excess β1 (1.06-fold) (Figure 1 left, middle panel). Its reduced level compared to the no-tetracycline samples (51% down with just tet and 72% down with both tet and the β1 plasmid) is the apparent competition of α1 with α3 that we are investigating.

Figure 1 (right) shows the response of cells expressing the α3 L924P mutation, one that we previously showed misfolds during biosynthesis and activates the unfolded protein response (UPR) [7,8]. Because of misfolding, the yield of α3 was lower in the mutant (24% lower than WT in this blot) and, in this case, no increase in β1 expression was seen when tetracycline turned on expression of α3 and transfected β1 subunits in L924P cells (1.02-fold). Instead, a faster-migrating form of β1 appeared with or without the β1 vector; this is the core-glycosylated form in the endoplasmic reticulum [7]. In previous work, we showed the redistribution of L924P Na,K-ATPase from plasma membrane to ER by sucrose density centrifugation [8]. The impact of misfolding on the cell was so great that α1 was redistributed in the sucrose gradient as well, which is a toxic cell biological consequence of the mutation. Here, both with and without the β1 plasmid, the immature band was 41–43% of the total β1. We speculate that the most likely reason for a smaller effect in L924P is impairment of biosynthesis followed by ER-associated degradation (ERAD) of misfolded complexes by proteasomes [22,23].

**Figure 2:**
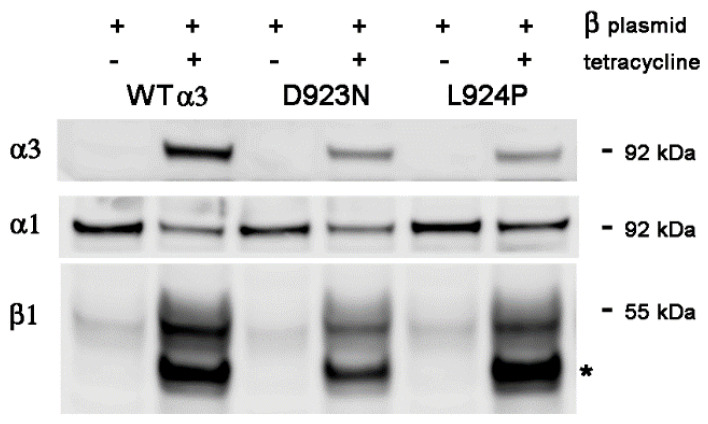
Transient overexpression of β1 for 48 h. This experiment differs from Figure 1 in that the cell line expressing the D923N mutation was included and a longer induction time after tetracycline addition was employed for the accumulation of newly made protein. A larger proportion of β1 was seen in the high mannose immature form; the exposure time was shorter than in Figure 1 to avoid overexposing it. The asterisk marks β1 with high mannose oligosaccharides. No significant increase in α1 was seen.

Figure 2 shows another transient transfection comparing α3WT, D923N, and L924P mutations where 48 h of tetracycline treatment allowed more time to accumulate α3 and the introduced β1. In this case, all samples had the β1 plasmid. It can be seen that, in the absence of induction of α3 and β1, the endogenous β1 migrates as a single fuzzy band at ~55 kDa, the form with mature glycosylation added in the Golgi apparatus. In the 24 h induction in Figure 1, a minor fraction of induced β1 appeared at the mobility of immature glycosylation of the ER (~40 kDa) but, after 48 h (Figure 2), the induced immature form was a major component even in the cells with WT α3. There are several alternative explanations for this observation: that the capacity of the cell to fully process β subunit has been exceeded; that the amount of α subunit needed for β to associate with in order to leave the ER is limiting; or perhaps that deleterious effects of lipofectamine limit the usefulness of transient transfections. Because of this ambiguity, further experiments employed cell lines with stable β1 transfection. We should note, however, that, as in Figure 1, the presence of excess β1 did not prevent the reduction in α1 level upon induction of α3. The reduction in α1 was 55% in WT, 47% in D923N, and 37% in L924P, similar to what we reported before [7]. Lower expression of α3 due to mutation correlates with less reduction in α1 regardless of the overexpression of β1.

### 2.3. Stable Transfection of β1 with α3WT

**Figure 3:**
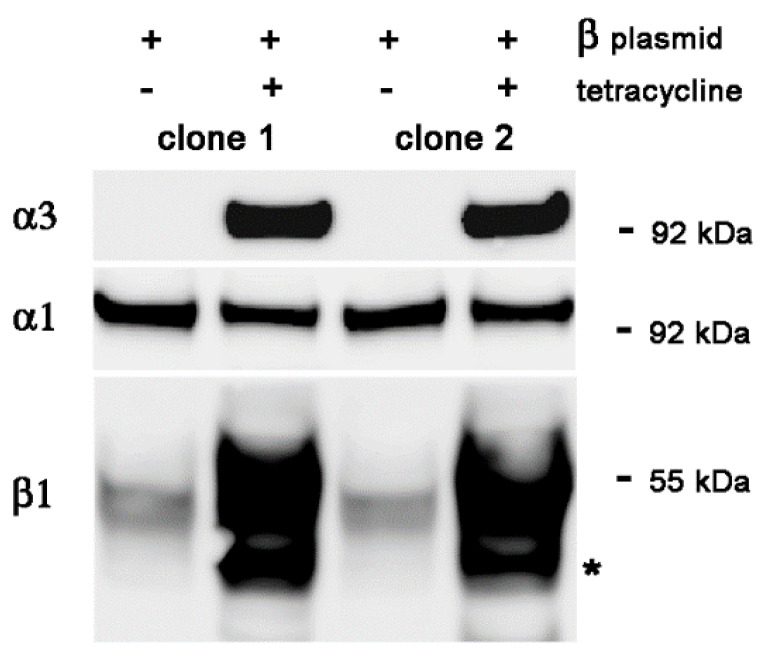
Stable lines expressing both exogenous α3 and exogenous β1 under tetracycline induction. Two independent stable cell lines with incorporated β1 plasmid DNA were made. They were grown with or without continuous tetracycline for more than four passages. Both lines gave robust induction of both α3 and β1. α1 levels were still reduced when α3 was turned on, and no independent increase in α1 was detected. The asterisk marks β1 with high mannose oligosaccharides.

**Figure 4:**
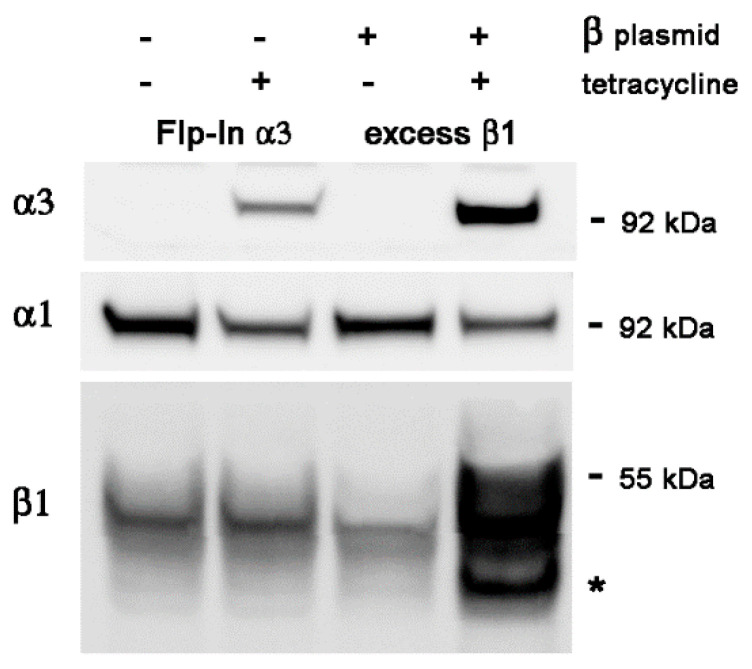
This experiment contrasts the response of the host WT α3-expressing Flp-In cell line with one of the derivative lines that also expresses excess β1. As before, α3 and α1 appeared to compete for expression, but expression of α1 was not rescued by the presence of excess β1. The asterisk marks β1 with high mannose oligosaccharides.

Starting with Flp-In 293 cells with WT human α3 cDNA at the FRT site, inducible by tetracycline, we selected for stable transfectants of WT human β1 cDNA, also inducible by tetracycline. Figure 3 shows two independent subclones of the α3WT-expressing Flp-In line with and without tet-induced expression of α3 and β1 together, 48 h after induction. As in Figure 2, there was dramatic induction of both genes, as well as substantial ER retention of excess β1 as shown by the presence of the faster-migrating form with core glycosylation. Again, reduction in α1 during α3 induction occurred (13% reduction in both clones). Figure 4 shows one of those excess β1 lines compared to the parental Flp-In line with and without tet induction of α3 and β1 together. In this case, there was greater expression of α3 with the excess β1 than without it (a 2.5-fold increase) but, again, there was still suppression of α1 expression (60% in the Flp-In parental line and 70% in the excess β1 line).

### 2.4. Stable Transfection of β1 with α3 Mutation L924P

**Figure 5:**
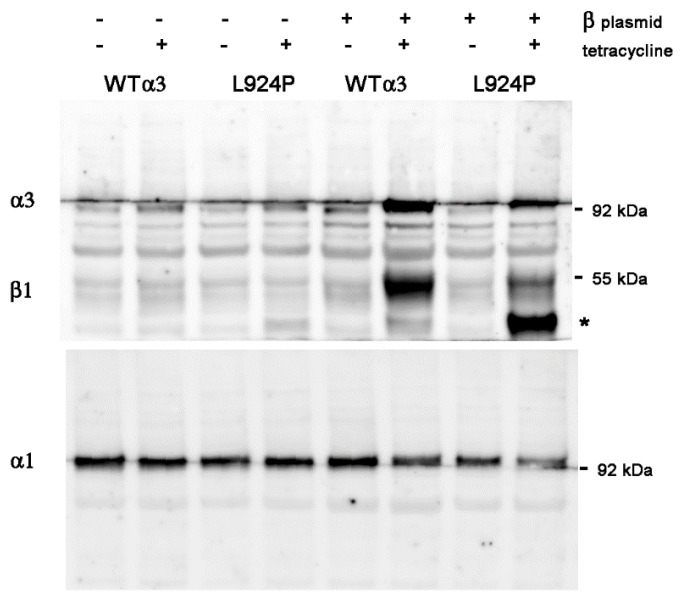
Contrast of the response of WT α3 and mutant L924P α3 with and without β1 plasmid. As always, two identical gels were run so that α3 and α1 could both be stained. Compared to the 24 h and 48 h transient transfections, the β1 subunit in continuous tetracycline induction was predominantly in the mature form for WT α3 and predominantly in the immature form for L924P. Nonetheless, the same results were obtained for the α subunits: increases in α3 and reductions in α1. The asterisk marks β1 with high mannose oligosaccharides.

Figure 5 compares the effect of β1 excess on α3 and β1 much as in Figure 1 but with and without stable transfection of β1. Tetracycline increased α3 in all samples, and the increase was larger in the lines with β1 transfection. For WT cells, the increase in α3 was 2.5-fold in the absence of extra β1 and 4.3-fold in its presence. As before, the levels of α3 were lower in the L924P cells than in α3WT cells and barely changed in the presence of excess β1. α1 levels were reduced by 13% in WT and not at all in L924P in the parent cell lines but were reduced 36% in WT and 11% in L924P with excess β1. Figure 5 confirms the findings of the transient transfections on β1 levels: they were 3.3-fold increased by tet induction of β1 expression, while the L924P mutant showed a 2.5-fold increase in mature β1 and a 10.3-fold increase in immature β1 when β1 was overexpressed.

**Figure 6:**
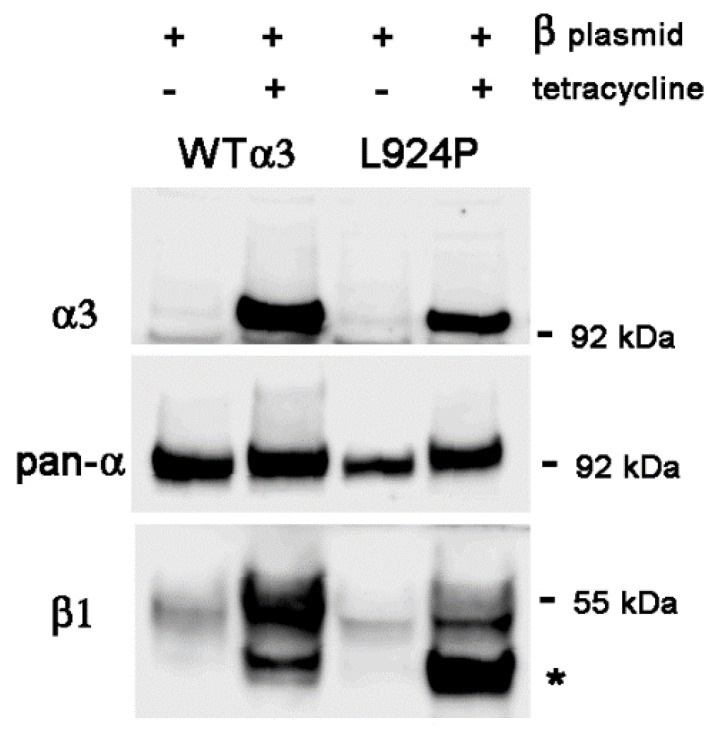
Total α subunit level in the presence of excess β1. This is a replicate experiment of Figure 5 but, instead of staining for α1, one blot was stained with a pan-α antibody. There was an increase in total α, but the results in all of the previous figures suggest that it was due to α3 and not to α1. The asterisk marks β1 with high mannose oligosaccharides.

Finally, Figure 6 repeats these findings, but the α subunits were stained by the α3-specific antibody in the top panel and a pan-specific antibody, 9A7, instead of the α1-specific antibody in the middle panel. There was a modest increase in total α subunit when both α3 and β1 were induced with tetracycline, 1.24-fold in WT and 1.23-fold in L924P, normalized to actin. In the presence of the β1 plasmid, the increase in β1 was 4.4-fold in WT and 3-fold in L924P, while the increase in immature β1 was 21-fold in both. This added up to a 6-fold increase in total β1 for WT and a 7-fold increase for L924P.

## 3. Discussion

### 3.1. Salient Findings

One advantage of the 293 Flp-In expression system is that the plasmid construct inserts into the genomic DNA at a single site, resulting in reproducible mRNA expression. We previously showed that α1 mRNA levels, measured by qPCR, did not change during the acute tetracycline induction of α3, while α3 mRNA levels rose as much as 40-fold over their low endogenous level [7]. We found no evidence for a compensatory reduction in α1 subunit transcription in 293 cells.

The experiment using a pan-specific Na,K-ATPase anti-α antibody indicated that the total of α protein increased by about 25% when α3 was induced [7]. Protein levels of α1 were decreased and α3 increased. The principal question was whether a rate-limiting supply of β subunit is responsible for the apparent competition of α1 and α3 when they are co-expressed. This is apparently not the case; a reduction in α1 persisted in all of the experiments, even when there was a large increase in β1. More importantly, there was no increase in α1 in any of the experiments. There was an increase in total α when tested, suggesting that excess β1 produced some net increase, but the data are consistent with a tetracycline-induced increase in α3 rather than a β-mediated increase in both α3 and α1.

Unlike in the Flp-In system, here, the stable transfection of a plasmid to express excess β1 was by antibiotic selection, and the plasmid may have inserted at more than one site. Prior work showed evidence of pools of Na,K-ATPase subunit in ER before assembly [24,25,26,27] associated with BiP or calnexin [18,28,29]. The presence of the immature glycosylated form here shows that our expectation of having an excess of β1 available in the ER was achieved. At the same time, the observation that a substantial fraction of excess β1 acquired mature glycosylation indicates that it was not entirely retained in ER. Others have also reported that an expressed β subunit apparently passed through the Golgi apparatus without an associated α subunit [30,31,32,33,34]. For Na,K-ATPase this apparently does not occur naturally. In the absence of α subunit, evidence was shown for an ER retention signal in the lumenal domain of β1 (but not in the lumenal domain of the H,K-ATPase β) [35]. The capacity of the protein that retains β1 (possibly chaperones like BiP and calnexin) may be exceeded when Na,K-ATPase β1 is greatly overexpressed.

### 3.2. Related Observations in the Literature

There have been other reports of reciprocal changes in Na,K-ATPase subunits. Transfection of monkey kidney cells with rat α1 resulted in no increase in activity or α1 protein despite higher mRNA levels, and it was speculated that β subunit was limiting [36]. Stable transfectants of HeLa cells with exogenous ouabain-resistant rat α1, α2, and α3 obtained from the Lingrel laboratory [37] were used to assess sodium fluxes, and it was reported that there was an apparently reciprocal reduction in the activity of the endogenous human α1 when the other α subunits were expressed [38]. In 2009, two other groups reported related findings. First, comparison of the levels of endogenous α and β subunits with overexpressed tagged subunits in stable HeLa and 293 cell lines showed reciprocal totals of α1 capped at the endogenous levels and reciprocal expression of β1 that could exceed endogenous β levels [39]. Second, endogenous α1 and transfected β1 and β2 subunits were similarly investigated in MDCK cells, with the conclusion that β levels were reciprocally affected, while the α1 level was fixed and apparently limiting [40]. A reduction in β3 protein when β1 was increased was also seen in a breast cancer cell line, while the level of β3 mRNA was, if anything, increased [41]. Finally, expression of 293 cell endogenous α1 and transiently expressed exogenous tagged α1 were followed spatially and quantitatively with immune detection and TIRF and super-resolution microscopy. The result was that, 41 h after transfection, total plasma membrane density of α1 was raised by 63%, while the endogenous α1 was reduced by 16% [42]. There, the conclusions were that transient expression can exceed endogenous levels but with some reduction in endogenous protein by competition. This caveat also affects the interpretation of our transient β1 transfections, but the proportions of α1 and α3 measured in our stable transfections should represent a true steady state after a few cell divisions. The consequences of competition for a limiting factor are also seen when the expression of an endogenous α subunit is reduced. The expression of α2-GFP and α3-GFP was substantially increased in H1299 cells when the endogenous α1 was silenced [43].

A similar phenomenon also appears to occur in animal models. In heterozygous mice with a loss-of-function allele of α3, an apparently compensatory 30% increase in α1 was reported in brain [44]. In our own work on the same mice (submitted), the level of α3 expressed in brain was 80% of WT, not the nominal 50%. Certain loss-of-function alleles (revertants with chromosomal inversions) of ATPα in *Drosophila* also resulted in greater than 50% expression of the unmodified allele [45]. There was 60–70%, not 50%, of skeletal muscle α1 expressed in heterozygous α1 knockout mice [46]. This apparently can occur with β subunit as well; in mice where β1 was knocked out in lung epithelial cells, β3 levels increased [47]. All of these reports (in cells or in animals) involved manipulation of the expression of either α or β, and all are consistent with capping of the total level of Na,K-ATPase during biosynthesis.

### 3.3. Unanswered Questions

#### 3.3.1. Involvement of the β3 Subunit

All of the Na,K-ATPase subunit α and β isoforms can substitute for each other in Xenopus oocytes [48], but more selective α–β associations may occur in mammalian cells, for example, α2 with β2 in the heart [49]. In tissues, α1 pairs with β1 in renal tubules and α3 with β1 in the brain, and β3 is not expressed in neurons. Transcriptomic and other investigations have shown that HEK293 cells are actually from an adrenal lineage, not kidney [50,51,52]. The adrenal gland has high β3 expression (data in GTeX; www.gtexportal.org, URL accessed on 26 June 2024).

We do not know if α1 has a preference for association with β1 or β3 when both are present or whether 293 cells possess any proteins that would regulate that association. However, even if α1 does not respond to elevated β1 because it is preferentially associated with β3, this still cannot explain the basic observation that α1 is reduced when α3 is increased. We can only conclude that the two α isoforms are competing for something else.

While β1 clearly can be overexpressed, we have not investigated whether that has an impact on endogenous β3. Do β3 and β1 compete for both α1 and α3? Is there a compensatory reduction in β3 when β1 is increased, like there is for α1 with α3 induction? β3 can be thought of as the mesenchymal β isoform. It was shown to be broadly expressed [53,54] and was first identified as a protein in lung, liver, testis, and skeletal muscle [55]. Only minor protein amounts were detected in brain, kidney, and heart, where Na,K-ATPase activity figures more prominently in physiology. A report showing high mRNA expression of β3 in the brain [53] was not confirmed by in situ hybridization in mouse brain in the Allen Brain Atlas, and ATP1B3 in the mouse and rat brain was later shown to be found in the cells with the least demand for ion transport, the oligodendrocytes [56]. β3 remains the most understudied component of Na,K-ATPase.

#### 3.3.2. Unknown Regulation of Degradation or Translation

A major feature of protein maturation in the ER is the degradation of excess or misfolded proteins [22,23], and it is to be expected that inhibition of ER-associated proteasomes will increase the level of Na,K-ATPase subunits, as we have seen with ATP1A3 mutations [8]. It cannot be ruled out that α1–α3 competition is mediated by ER-related degradation pathways. The present approach also does not rule out selective degradation of mature, plasma-membrane-associated α1, such as by ubiquitination [57], although it seems unlikely.

Regulation of translation by means other than competition for a limiting factor has also not been ruled out. Early in vitro translation studies using reticulocyte, pancreatic, or wheat germ lysates showed that α and β subunits were translated separately and that their co-translational assembly could be somewhat delayed [10,13,58] and occurred only in the presence of microsomes [11,24,59]. When crystal structures of Na,K-ATPase were obtained, it became clear that the α–β interaction surfaces were available only late in translation, so the slow assembly did not have to be due to regulation of translation per se. Direct investigation of translation gave evidence of reduced efficiency of translation of Na,K-ATPase α and β subunits involving 5′UTR or 3′UTR mRNA sequences and possibly involving microRNAs [60,61,62,63]. In another case, MDCK cells that had been dedifferentiated by Maloney sarcoma virus (MSV) transformation expressed reduced levels of β1. Expression of exogenous β1 mRNA increased α translation efficiency, and initial translation rates were 6-7-fold higher [64]. It should be borne in mind, however, that, in our experiments, only the endogenous α1 and endogenous β1 and β3 have their full 5′UTR and 3′UTR available for interaction with translational regulation factors, since the α3 and excess β1 plasmid constructs are built with coding regions only.

We speculate that a chaperone or combination of chaperones required for assembly is rate-limiting and that it recognizes α1 and α3 more or less equally so that they compete. Since the largest α–β contact area is at the ER lumenal surface, the ER chaperones ERdj3, BiP, and Grp94 (analogs of the cytoplasmic chaperones Hsp40, Hsp70, and Hsp90) could be involved and may involve transfer of α subunit from one to another. It is expected that unassembled α subunit is then guided by the ERAD disposal pathway, using ERAD adaptors, retrotranslocation, ubiquitination, and proteasomal degradation. It appears that control of β subunit is less stringent, since it demonstrably accumulated in the immature glycosylated form here. We do not currently have a way to detect the status of α subunits alone in the ER.

The field of translation regulation has advanced, but recent research on Na,K-ATPase biosynthesis is lacking. The observations most similar to ours, made in both MDCK and 293 cells, were interpreted as evidence for translational repression [39]. Their expression constructs were also without UTR sequence, and the authors stressed that the effects seen must be due to the coding sequences of the down-regulated polypeptides [39]. We cautiously propose that the subunit interactions described here and in Clifford et al. 2009 [39] are post-translational in the sense of “after ribosome translation” but early in protein maturation in the ER.

## 4. Materials and Methods

The experimental system is stable human HEK293 cell lines generated with the Flp-In system (Flp-In™ T-REX™ 293 cells, Life Technologies, Carlsbad, CA, USA), as previously reported [9]. The host cell line, which is not an over-expressor like HEK293T, was engineered to have a single FRT recombination site in a transcriptionally active location. Individual mutations were made in a plasmid that has ATP1A3 cDNA under a tetracycline-induced promoter. The plasmid also has an FRT site and recombination at the cell’s FRT site in the genomic DNA was achieved with a helper plasmid, pOG44, and antibiotic selection. The host cell line expresses the endogenous human ATP1A1 subunit, but endogenous ATP1A3 is expressed at negligible levels when grown in tetracycline-free 10% fetal bovine serum. Exogenous ATP1A3 is induced with continuous tetracycline, 1 μg/mL. All of the human Na,K-ATPase α subunits are sensitive to ouabain, but the ATP1A3 cDNA in the plasmid has two substitutions adopted from the naturally ouabain-resistant mouse α1, Q108R and N119D [65]. In experiments, ouabain at low concentrations inhibits α1 and kills the cells, but it does not inhibit the introduced ouabain-resistant α3 when its expression is turned on with tetracycline. If the α3 has enough activity, the cells survive. In this way, the survival test is often used as a first test of the pathogenicity of new Na,K-ATPase mutations.

To overexpress β1 subunit transiently, two different plasmids were used: pcDNA4/TO/b1 expression vector harboring human β1 cDNA and tet operator (generous gift of Dr. J.H. Kaplan, University of Illinois at Chicago, Chicago, IL, USA) and pcDNA6/TR plasmid (Invitrogen, Waltham, MA, USA) that constitutively expresses the tet-sensitive repressor protein under the control of the human CMV promoter. The 293/Flp-In cells expressing either WT α3 subunit or the mutants L924P and D923N were co-transfected with those plasmids at a ratio of 1:5, respectively, using the lipofectamine protocol. After 24 h recovery, the cells were induced with tetracycline, 1mg/mL, for 24–48 h at 37 °C. Cell lysis was performed in RIPA buffer containing protease inhibitor cocktail (Roche Diagnostics, Indianapolis, IN, USA). Inducible expression of β1 was tested by Western blot.

To generate stable clones overexpressing β1 subunit, cells were co-transfected with pcDNA4/TO/b1 and pcDNA6/TR plasmids, as stated above, split 48 h post-transfection and exposed to Zeocin for selection, 250–500 mg/mL. Host 293/Flp-In/α3 cells are Zeocin-sensitive. Resistance to antibiotic was conferred by expression of the pcDNA4/TO/b1 vector. Two other antibiotics, blasticidin (10 mg/mL) and hygromycin (100 mg/mL) (InVivogen), were present in the growth medium to support expression of the α3 constructs. Induction of both β1 and α3 subunits was with tetracycline, 1 mg/mL.

### Detection of Na,K-ATPase Subunits

Gel electrophoresis and Western blots were performed using Invitrogen NuPage 4–12% MES gels and transfer onto nitrocellulose membranes. α3 subunit of Na,K-ATPase was detected with the goat peptide-specific polyclonal antibody C16 or with the mouse monoclonal antibodies, clones H4 or F1 (all from Santa Cruz Biotechnology, Dallas, TX, USA). The 9A7 pan-specific α subunit antibody was a generous gift of Maureen McEnery, Case Western Reserve School of Medicine. Monoclonal antibodies 6F (Developmental Studies Hybridoma Bank, University of Iowa, Iowa City, IA, USA) or M17-P5-F11 (generous gift of Dr James Ball, University of Cincinnati, Cincinnati, OH, USA) were used to detect human α1 and β1 subunits of Na,K-ATPase, respectively. Loading controls were anti-actin-HRP (Sigma, St. Louis, MO, USA). Signal was developed with chemiluminescence (WesternBright ECL, Advansta, San Jose, CA, USA) and images were collected with an LAS 4000 imager (GE Healthcare, Chicago, IL, USA).

## Data Availability

All of the data are within the article.
